# Attitudes and practices to adult vaccination among physicians before and after COVID-19 pandemic in the United Arab Emirates

**DOI:** 10.1016/j.jvacx.2024.100455

**Published:** 2024-02-01

**Authors:** Hiba J. Barqawi, Kamel A. Samara, Enad S. Haddad, Layane M. Bakkour, Firas B. Amawi

**Affiliations:** a Department of Clinical Sciences, College of Medicine, University of Sharjah, United Arab Emirates; b College of Medicine, University of Sharjah, Sharjah, United Arab Emirates; c Dr. Sulaiman Al Habib Hospital, Dubai, United Arab Emirates

**Keywords:** Adult vaccination, Vaccine attitudes, Vaccine practices, Preventive practices, Influenza, Vaccine preventable diseases

## Abstract

**Introduction:**

Vaccination remains underutilised worldwide with low vaccine uptake rates across the board with many adults remaining unprotected. Across the Arab world, attitudes towards vaccines vary but high rates of vaccine hesitancy have been found. This study aims to explore the adult vaccination attitudes and practices by physicians in the UAE, both before and after the introduction of the COVID-19 vaccines.

**Methodology:**

This cross-sectional, descriptive study used convenience and snowball sampling to collect comprehensive data from UAE physicians. A self-administered questionnaire was distributed in two stages: the first (pre-COVID-19 vaccines) between the months of June and October 2020 and the second between the months of November 2022 and March 2023.

**Results:**

1000 responses, 500 from each time period, were collected. Nearly a third were family physicians or internists with more than 70% of the physicians working in governmental hospitals. 95% agreed that vaccines are safe in both cohorts but 74.4% reported not having enough time to advise about vaccines. 80.8% of physicians in the 2022 cohort reported safety concerns as the most common reason for patients to refuse vaccines. The most recommended vaccines were influenza (68.6%), Hepatitis B (66.0%) and HPV (61.4%), with pneumococcal coming in close at 57.8%. Family medicine physicians showed the highest utilisation of preventive practices across both cohorts. Nearly half of all family medicine physicians did not regularly evaluate both the influenza and general immunisation status of their patients. 54.6% of physicians reported having patients with VPDs in the last five years (not including COVID-19) in 2022.

**Conclusion:**

Physicians have overly positive attitudes, but their practices reflect a more superficial appreciation of vaccines and lack of initiative. Physicians need to adopt a pro-vaccine stance, armed with the proper tools and the right mentality and beliefs.

## Introduction

Being one of public health’s greatest achievements, vaccination remains underutilised in all corners of the globe. Behind this lies no simple reason, but some combination of a lack of availability, non-negligible level of distrust, lax immunisation laws, and structural limitations [8]. Even before COVID-19 had brought vaccines to the top of every local and international health body’s docket, the WHO had already classified vaccine hesitancy, defined as the reluctance or refusal to vaccinate despite availability, as one of the top ten threats to global health [35]. Yet, vaccine hesitancy has been apparent in global research for the last twenty years [13]. The situation gets even more dismal when discussing adult and elderly vaccination, with a $7.1 billion economic burden attributed to unvaccinated adults contracting Vaccine Preventable Diseases (VPDs) in the United States (US) [19]. There, adult vaccination rates are still suboptimal, with low vaccine uptake rates across the board with many adults remaining unprotected according to the most recent Centers for Disease Control and Prevention (CDC) Surveillance report [14]. Moreover, healthcare providers (HCPs) play a critical role in fostering vaccine acceptance among those that are hesitant [34], yet varying levels of scepticism and lack of leadership and vaccine promotion have been documented among them [30].

As such, a huge effort has been undertaken to delineate the various causes of hesitancy and methods to address it. Fundamentally however, most strategies have education as one of their essential blocks; in a recent systematic review by Singh et al., “most of the interventions … were primarily either to inform or educate the target population,” [29]. Physicians play a key role in patient education, especially when it comes to vaccines and combatting disinformation; conversely, their knowledge gaps, poor attitudes and practices have been found to negatively impact vaccine uptake [3], [9], [11], [13], [16], [18], [20], [21], [25], [32]. In a previous study, the authors had already quantified the level of knowledge regarding adult vaccination in the United Arab Emirates (UAE), finding poor knowledge regarding the adult vaccination schedule coupled with unclear and outdated guidelines [5].

A large amount of research during the COVID-19 pandemic also was dedicated to exploring the attitudes and practices towards the COVID-19 vaccines; results varied significantly, only one of four Saudi Arabia physicians would have agreed to take the vaccines in the early stages [23]). A multinational study showed hesitancy rates among physicians reaching as high as 32.8% across the Arab World, with concerns about side effects and distrust regarding expedited vaccine production being the most cited reasons [24]. In the UAE, only 24 out of 135 surveyed HCWs had unconditional acceptance of the COVID-19 vaccine [24]. Yet, another more focused study by AlKetbi et al. found an 89.2% acceptance rate for COVID-19 vaccines among the HCWs, highlighting a level of discrepancy between reported results but the existence of some non-negligible level of vaccine hesitancy [1]. Regarding general vaccination attitudes, a single UAE qualitative study was conducted by Elbarazi et al. exploring vaccine hesitancy; they found positive attitudes but poor practices and empowerment to deal with vaccine hesitancy [6]. While the current UAE population is characterized as young, with less than 1.3% of the population being below the age of 65, this is expected to increase eighteenfold by 2050, reaching 18.6% [2]. This growth is expected to shift priorities in healthcare services as well as lead to rising healthcare costs. As such, this study aims to explore and elucidate gaps in the UAE physicians’ adult vaccination attitudes and practices, both before and after the introduction of the COVID-19 vaccines.

## Methodology

### Study population

This cross-sectional, descriptive study was used to collect comprehensive data from UAE physicians all over the country in two stages. The first involved data collection between 1st June and 5th October 2020, before any COVID-19 vaccination campaigns; the second stage occurred between 7th November 2022 and 30th March 2023, during which COVID-19 vaccination campaigns were wrapping up with vaccine coverage nearing 100% in the country. This timeframe was expected to allow for an evaluation of vaccination attitudes and practices both before and after the national COVID-19 vaccination campaign. Convenience and snowball sampling was used with participants being approached through email, phone, Whatsapp, and social media. Inclusion criteria for this study included being a physician, having at least 1 year of work experience, and currently practising medicine in the UAE. Care was taken to target every department, health authority, and levels of training to ensure all groups are represented. The minimum required sample size was found to be 385 participants using Cochran’s sample size formula using a confidence level of 95%, sampling error of 5%, and a standard error of 1.96. The number was increased by 20% to account for non-response. A participant information sheet (PIS) was presented before starting the questionnaire, and written consent obtained prior to participation in the study. Finally, the collected data was available only to the investigators to ensure confidentiality, and no identifying data was collected.

### Questionnaire development

A 43-question, 73-item, self-administered questionnaire consisted of three different sections: demographics, general and adult vaccination attitudes, and adult vaccination practices. The 2020 questionnaire also consisted of ten additional questions related to specific vaccine attitudes that were not included in the 2022 questionnaire. 4-item Likert scales, multi-select questions, as well as single-choice questions were used. The questionnaire was developed based on the vaccination and preventive practices literature. Only an English version was developed and pilot tested two times, with each pilot involving fifteen practicing physicians in the UAE from different departments and levels of training; all provided feedback was evaluated and incorporated if appropriate. This research was reviewed and approved by the Research Ethics Committee of the University of Sharjah (Reference Number: REC-20–04-09–01-S). It was conducted in accordance with all relevant guidelines and regulations.

### Statistical analysis

Data was exported from Google Forms to CSV format and processed in python-3 using the Matplotlib, pandas, statspy and statsmodels packages for analysis and interpretation. Missing values were dealt with through pairwise deletion. Frequency distributions were calculated for categorical variables and reported percentages were calculated by excluding the missing values. No outliers were detected. An average vaccine attitudes score was calculated from the items related to physician vaccine attitudes followed by binning into three groups: excellent attitudes (score ≥ 90%), good attitudes (score ≥ 75%), and hesitant attitudes (score < 75%). The non-parametric Mann-Whitney *U* test was used to compare between scores before and after COVID-19 where applicable. All demographic and professional variables, average vaccination attitudes, and evaluation of influenza status were evaluated as predictors of evaluating general immunization status. Bivariate analyses were conducted to identify significant predictors using chi-squared tests. The significant predictors were then fed into a multivariate logistic regression model, which was evaluated using a log-likelihood ratio test. P values less than 0.05 were taken to be significant.

## Results

### Demographics of participants

Overall, from a total of 2,200 questionnaires distributed, 1042 responses were collected, yielding a 47.3% response rate, in line with global averages for physician studies [17]. For the data collected in 2020 (henceforth referred to as the 2020 cohort), 534 questionnaires were collected out of which 34 did not meet inclusion criteria, resulting in a total of 500 participating physicians. As for the 2022 data (henceforth referred to as the 2022 cohort), 508 questionnaires were collected, with eight not meeting inclusion criteria, funnily enough leading to another 500 participating physicians in the second cohort. The demographics of both cohorts can be seen in Table 1. 12.4% of the 2022 cohort had also participated in the 2020 cohort. A female predominance can be seen in both cohorts. However, the 2022 cohort is characterised by a lower representation of Emirati physicians, as well as lower proportion of family medicine doctors. The EHS (Emirates Health Services) was the most represented health authority (which is responsible for all the Emirates with the exception of Dubai and Abu Dhabi), though the other two authorities were also well-represented across both cohorts. More than 70% of the physicians worked in governmental hospitals in both cohorts and saw more than 20 patients a week.

**Table 1:** Demographics of both cohorts; MOHAP: Ministry of Health and Prevention (which represents Sharjah and the Northern Emirates); DHA: Dubai Health Authority (which represents the Emirate of Dubai); DOH/HAAD/SEHA: Department of Health (which represents the Emirate of Abu Dhabi).

Feature	Number (%) – 2020 Cohort	Number (%) – 2022 Cohort
**Sex**	(n = 500)	(n = 500)
Female	n = 298 (59.6%)	n = 282 (56.4%)
Male	n = 202 (40.4%)	n = 218 (43.6%)
**Citizenship**	(n = 500)	(n = 500)
Other Arab	n = 304 (60.8%)	n = 329 (65.8%)
Emirati	n = 115 (23.0%)	n = 60 (12.0%)
Non-Arab	n = 81 (16.2%)	n = 111 (22.2%)
**Department**	(n = 364)	(n = 365)
Internal medicine	n = 88 (24.2%)	n = 99 (27.1%)
Family medicine	n = 86 (23.6%)	n = 43 (11.8%)
Pediatrics	n = 42 (11.5%)	n = 42 (11.5%)
Others	n = 148 (40.7%)	n = 181 (49.6%)
**Health Authority**	(n = 498)	(n = 487)
MOHAP	n = 184 (36.9%)	n = 207 (42.5%)
DHA	n = 161 (32.3%)	n = 172 (35.3%)
DOH/HAAD/SEHA	n = 153 (30.7%)	n = 108 (22.2%)
**Level of Training**	(n = 500)	(n = 500)
Intern house officer	n = 134 (26.8%)	n = 135 (27.0%)
Resident	n = 153 (30.6%)	n = 145 (29.0%)
General practitioner	n = 82 (16.4%)	n = 51 (10.2%)
Specialist	n = 53 (10.6%)	n = 72 (14.4%)
Senior specialist	n = 18 (3.6%)	n = 35 (7.0%)
Consultant	n = 60 (12.0%)	n = 62 (12.4%)
**Workplace**	(n = 500)	(n = 499)
Government hospital	n = 356 (71.2%)	n = 361 (72.3%)
Private clinic/hospital	n = 90 (18.0%)	n = 102 (20.4%)
Primary healthcare	n = 54 (10.8%)	n = 36 (7.2%)
**Number of patients seen in a week**	(n = 500)	(n = 500)
1 to 19	n = 110 (22.0%)	n = 96 (19.2%)
20 to 49	n = 210 (42.0%)	n = 208 (41.6%)
50 and above	n = 180 (36.0%)	n = 196 (39.2%)

### General adult vaccination attitudes

Table 2 presents the general adult vaccine attitudes for both cohorts. Cronbach’s alpha for this subscale was found to be 0.73, with the 95% confidence interval being [0.71, 0.76], indicating it had acceptable internal consistency. Nearly 95% agreed that vaccines are safe in both cohorts. A significant rise can be seen in the view that vaccinating adults is important with the number agreeing with the statement increasing to 92.4% in 2022. Similarly, the benefits of vaccines were viewed to outweigh the harm in both cohorts with no significant change. However, 74.4% reported not having enough time to advise about vaccines and another two-thirds discussing how physicians tend to focus more on treatment rather than prevention. When discussing reasons a patient might refuse a vaccine generally, safety concerns were the most agreed upon, with a significant rise in 2022 (80.8%) compared to 2020 (59.4%). Other significant increases were seen with regards to efficacy concerns (rising to 54.6% from 43.4%), and religious concerns (rising to 20% from 14.2%), while cost was identified less as a barrier in 2022, having dropped to 28.4% from 46.8%. Still a large majority believed that vaccination rates are low due to a lack of legal mandates and more than 90% agreed that national adult vaccination and awareness campaigns are needed. In addition, a lack of coverage of vaccines under insurance plans was less identified as a barrier to adult vaccination, though 61.4% of physicians in 2022 identified it as such. Finally, 54.8% of physicians reported more positive attitudes to vaccination during the COVID-19 pandemic (with only 6.0% reporting more negative attitudes); patients’ attitudes also mirrored this, with 49.0% of physicians reporting patients having more positive attitudes (compared to 20.4% developing more negative ones).

**Table 2:** Vaccination attitudes for both cohorts. Mann-Whitney *U* test was conducted to evaluate whether there was a significant difference in attitudes after the national COVID-19 vaccination campaigns. * indicates a significant P value.

**Statement**	**Cohort (n = 500 for both)**	**Strongly disagree**	**Disagree**	**Agree**	**Strongly agree**	**P value**
**Vaccines are safe.**	2020	0.6%	4.0%	40.8%	54.6%	0.062
	2022	0.8%	4.2%	34.0%	61.0%	
Vaccinating adults is important.*	2020	1.4%	11.0%	34.8%	52.8%	<0.0005*
	2022	0.8%	6.8%	24.8%	67.6%	
**The benefit of all recommended vaccines outweighs their harm.**	2020	1.0%	6.8%	33.4%	58.8%	0.467
	2022	1.4%	8.4%	28.0%	62.2%	
There is often a lack of time for advising about vaccines.*	2020	5.4%	23.2%	43.0%	28.4%	0.047*
	2022	6.4%	19.2%	38.8%	35.6%	
**Physicians focus more on treatment rather than prevention.**	2020	7.0%	24.4%	39.2%	29.4%	0.959
	2022	9.8%	23.6%	33.8%	32.8%	
**Patients refuse recommended vaccines when they are healthy.**	2020	4.6%	27.0%	44.4%	24.0%	0.962
	2022	5.4%	27.8%	41.0%	25.8%	
Patients refuse vaccines due to safety concerns.*	2020	7.2%	33.4%	42.4%	17.0%	<0.0005*
	2022	4.2%	15.6%	52.8%	27.4%	
Patients refuse vaccines due to efficacy concerns.*	2020	15.8%	40.8%	32.8%	10.6%	<0.0005*
	2022	12.4%	33.0%	37.2%	17.4%	
Patients refuse vaccines due to cost.*	2020	22.6%	30.6%	29.8%	17.0%	<0.0005*
	2022	45.4%	26.2%	18.0%	10.4%	
Patients refuse vaccines if they are not covered under insurance.*	2020	4.8%	15.4%	39.2%	40.6%	<0.0005*
	2022	16.0%	19.8%	32.2%	32.0%	
Patients refuse vaccines due to religious concerns.*	2020	53.4%	32.4%	10.8%	3.4%	0.003*
	2022	45.2%	34.8%	13.6%	6.4%	
**Patients refuse a vaccine because they think they will not get the disease.**	2020	9.8%	26.8%	46.0%	17.4%	0.260
	2022	9.8%	24.8%	44.2%	21.2%	
**Adult vaccination rates are low because vaccines are not legally mandatory.**	2020	6.2%	18.4%	44.4%	31.0%	0.573
	2022	8.0%	22.6%	35.4%	34.0%	
Adult vaccination rates are low because vaccines are not covered under some insurance plans.*	2020	4.6%	17.4%	42.8%	35.2%	<0.0005*
	2022	13.2%	25.4%	35.0%	26.4%	
**There should be more national adult vaccination and awareness campaigns.**	2020	1.8%	5.6%	29.6%	63.0%	0.245
	2022	2.0%	6.8%	31.6%	59.6%	

### Specific adult vaccination attitudes

**Fig. 1:**
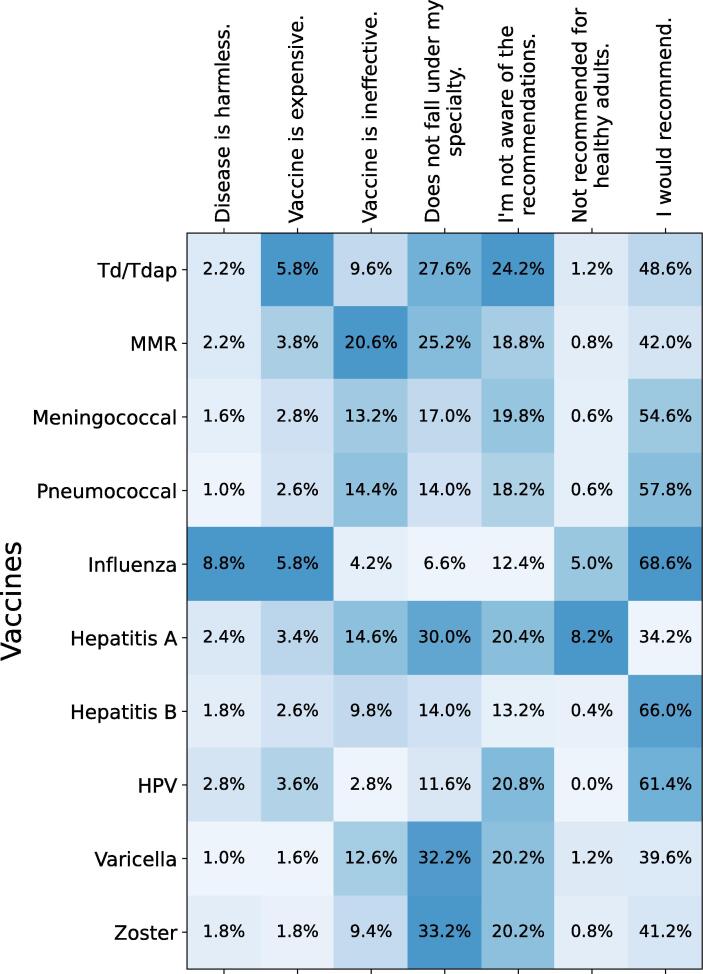
The reasons a participant physician may not recommend a specific vaccine. This data was only collected for the 2020 cohort. Color coding is done on a column-by-column basis.

The 2020 cohort were asked about reasons they may not recommend specific adult vaccines but no such questions were included in the 2022 cohort. Fig. 1 represents the results for the various vaccines and reasons. The most recommended vaccines were influenza (68.6%), Hepatitis B (66.0%) and HPV (61.4%), with pneumococcal coming in close at 57.8%. For Td/Tdap, MMR, Hepatitis A, Varicella, and Zoster, the most common reason for not recommending them was the vaccine not falling under the physician’s specialty. A lack of knowledge regarding recommendations was also among the most common reasons for not recommending any of the vaccines surveyed. Interestingly, influenza was by far the most likely to be regarded as a harmless disease, nearly four-times the closest contender (Hepatitis A). Its vaccine was also the most commonly cited as expensive (along with Td/Tdap) at 5.8% of responses.

### Preventive practices

**Fig. 2:**
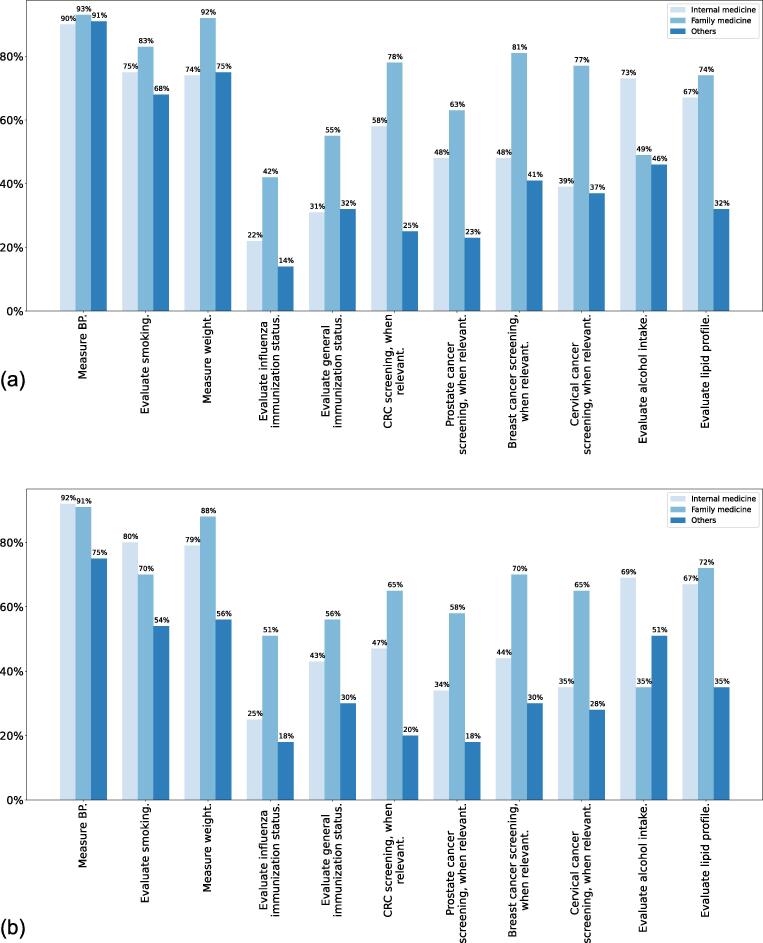
The preventive practices done by a physician. (a) shows the results for the 2020 cohort while (b) shows the results for the 2022 cohort.

Both cohorts were asked about what preventive practices they would regularly perform in their clinic. Fig. 2 represents the results for the 2020 and 2022 cohorts. Family medicine doctors showed the highest utilisation of preventive practices across both cohorts. Yet, nearly half of all family medicine physicians did not regularly evaluate both the influenza and general immunisation status of their patients. Similarly, for internal medicine doctors, only one in four evaluated the need for influenza vaccination while less than half looked at the general vaccination status (though it had increased substantially from only a third in 2020). Multivariate analysis showed that being female (p = 0.033, OR = 1.391 (95% CI: 1.027–1.885)), paediatrician (p < 0.0005, OR = 5.882 (95% CI: 3.034–11.404)), general practitioner (p = 0.036, OR = 1.780 (95% CI: 1.038 – 3.053)), and evaluator of influenza immunization status (p < 0.0005, OR = 6.439 (95% CI: 4.609 – 8.998)) to all predict higher likelihood of evaluating general immunization status. No difference was found attributable to health authority, number of patients seen in a week, or before and after the pandemic. All multivariate results are displayed in Table 3.

**Table 3:** The results of the logistic regression modeling the evaluation of general immunization and exploring its determinants. P values for the bivariate chi-squared tests are reported below each variable. Rows with significant p values are bolded. OR: odds ratio; CI: confidence interval; SE: standard error; MOHAP: Ministry of Health and Prevention; DHA: Dubai Health Authority; DOH/HAAD/SEHA: Department of Health.

**Evaluating General Immunization Status – Binary Logistic Regression (LR)**						
**Model Terms**		$\text{OR}(e^{\beta_{i}})$	**95% CI for OR**	**SE**	**z-Statistic**	**P value**
**Intercept (**$\beta_{0}$**)**		**0.262**	**0.148**–**0.465**	**0.293**	**−4.573**	**<0.0005**
**Sex** **(P value: 0.001)**	Male	-	-	-	-	-
	**Female**	**1.391**	**1.027**–**1.885**	**0.155**	**2.133**	**0.033**
**Department** **(P value: < 0.0005)**	Others	–	–	–	–	–
	Family medicine	1.302	0.791–2.143	0.254	1.038	0.299
	Internal medicine	1.174	0.776–1.774	0.211	0.760	0.447
	**Pediatrics**	**5.882**	**3.034**–**11.404**	**0.338**	**5.247**	**<0.0005**
**Health Authority** **(P value: 0.019)**	MOHAP	–	–	–	–	–
	DHA	1.009	0.706–1.442	0.182	0.050	0.960
	DOH/HAAD/SEHA	1.134	0.770–1.669	0.197	0.638	0.524
**Average Vaccine Attitudes** **(P value: 0.042)**	Hesitant	–	–	–	–	–
	Good	0.924	0.553–1.545	0.262	−0.302	0.763
	Excellent	1.047	0.645–1.699	0.247	0.185	0.853
**Level of Training** **(P value: 0.005)**	Intern house officer	–	–	–	–	–
	Resident	0.994	0.636–1.553	0.228	−0.029	0.977
	**General practitioner**	**1.780**	**1.038**–**3.053**	**0.275**	**2.095**	**0.036**
	Specialist	0.633	0.371–1.080	0.273	−1.676	0.094
	Senior specialist	1.056	0.515–2.164	0.366	0.148	0.883
	Consultant	0.868	0.502–1.499	0.279	−0.508	0.611
**Evaluating Influenza Immunization Status** **(P value: < 0.0005)**	No	–	–	–	–	–
	**Yes**	**6.439**	**4.609**–**8.998**	**0.171**	**10.919**	**<0.0005**
**Log-Likelihood: −556.41**	**Log-Likelihood of Null Model: −677.22**		**Log-Likelihood Ratio P value: <0.0005**			

The most performed practices were measuring blood pressure and weight as well as evaluating smoking. Cancer screening and alcohol intake evaluation both appear to be severely underutilised by the majority physician’s surveyed with utilisation rates as low as 18% for some of the practices. Family physicians showed highest usage of preventative practices except in alcohol usage where internists evaluated at a rate of 69% compared to family physicians at 35% in 2022.

### Vaccination practices

The physicians in both cohorts were asked a number of questions related to their vaccination practices and patient education. Table 4 shows the results for both cohorts. 54.6% of physicians reported having patients with VPDs in the last five years (not including COVID-19) in 2022 (a minor rise from 52.8% in 2020). Family physicians were identified as having the most responsibility for determining a patient's immunisation status at 91.8% in 2022 (compared to 93.8% in 2020), followed by internists at 46.4% and then nurses at 31.0%, with similar results in 2020. Verbal discussion was the most common method for evaluating the immunisation status in 2022 (70.8%) and 2020 (72.4%). The majority of physicians also depended on checking the patient's file (∼60%) and the immunisation records (∼40%). Only 16.2% reported not determining the immunisation status at all. Interestingly, only 18.2% viewed it as their responsibility to determine the patient’s immunisation status, with more than a third disagreeing. Moreover, doctor’s screening for VPDs as well as their perceptions of other doctor’s screening was quite low: 35.0% (up from 31.6%) rarely screened their patients with 33.8% (down from 40.2%) only sometimes screening their patients in the 2022 cohort. Similarly, three-fourths of the physicians believed others also rarely or only sometimes screened their patients. Finally, the majority of physicians in the 2022 cohort reported moderate difficulty with evaluating a patient's immunisation status (39.8%), with another 34.6% finding some difficulty (results similar to those in 2020 as well).

**Table 4:** Vaccination practices. N.A.: Not Applicable (No COVID-19 vaccine was available during the surveying of the first cohort).

**Who should be responsible for determining the patient's immunization status?**			**Which of the following do you use to evaluate the patient's general immunization status?**		
**Option**	**2020**	**2022**	**Option**	**2020**	**2022**
Internal Medicine doctors	48.6%	46.4%	I verbally ask the patient.	72.4%	70.8%
Family Medicine doctors	93.8%	91.8%	Other medical staff ask the patient.	18.0%	16.2%
Emergency doctors	18.6%	17.0%	I check the patient's file.	62.6%	59.0%
Obstetricians and Gynecologists	23.0%	20.2%	I check the patient’s immunization records.	47.8%	39.8%
Nurses	33.8%	31.0%	I do not determine the immunization status.	17.6%	16.2%
Pharmacists	3.2%	6.2%			
**Have you had ANY patients with a vaccine preventable disease in the last 5 years?**					
**Cohort**	Yes	No		I cannot recall	
**2020**	52.8%	17.0%		30.2%	
**2022**	54.6%	18.8%		26.6%	
**To what extent does the responsibility of determining the patient's immunization status fall on you?**					
**Cohort**	1 (Not at all)	2	3	4 (Completely)	
**2020**	18.6%	23.0%	43.6%	14.8%	
**2022**	14.8%	24.4%	42.6%	18.2%	
**How difficult is it to evaluate the patient's immunization status?**					
**Cohort**	1 (Not at all)	2	3	4 (Very difficult)	
**2020**	12.8%	36.0%	39.4%	11.8%	
**2022**	15.4%	34.6%	39.8%	10.2%	
**How often do you screen your patients for vaccine preventable diseases?**					
**Cohort**	1 (Rarely)	2	3	4 (Always)	
**2020**	31.6%	40.2%	21.2%	7.0%	
**2022**	35.0%	33.8%	24.6%	6.6%	
**How often do you think other doctors screen their patients for vaccine preventable diseases?**					
**Cohort**	1 (Rarely)	2	3	4 (Always)	
**2020**	22.0%	52.0%	22.6%	3.4%	
**2022**	30.4%	43.0%	22.2%	4.4%	
**In a month, how many would REFUSE the influenza vaccine when recommended?**					
**Cohort**	0% to 24%	25% to 49%	50% to 74%	75% to 100%	Vaccine does not fall under my department.
**2020**	28.0%	19.6%	11.0%	2.0%	39.4%
**2022**	17.8%	23.6%	14.4%	2.2%	42.0%
**In a month, how many would REFUSE the COVID-19 vaccine when recommended?**					
**Cohort**	0% to 24%	25% to 49%	50% to 74%	75% to 100%	Vaccine does not fall under my department.
**2020**	N.A.	N.A.	N.A.	N.A.	N.A.
**2022**	37.4%	15.0%	8.6%	2.0%	37.0%
**In a month, how many would REFUSE a non-influenza/COVID-19 vaccine when recommended?**					
**Cohort**	0% to 24%	25% to 49%	50% to 74%	75% to 100%	Vaccine does not fall under my department.
**2020**	26.8%	15.6%	12.2%	3.6%	41.8%
**2022**	20.4%	19.4%	13.6%	3.8%	42.8%

Physicians were asked about the rates at which various vaccines would be refused when recommended, the results of which are found in Table 4. The most common response for all three vaccine groups (Influenza vaccine, COVID-19 vaccine, non-Influenza-non-COVID-19 vaccine) was that the vaccine does not fall under the physician’s specialty. For those that did recommend vaccines, refusal rates differed based on the cohorts and vaccine. Influenza refusal rates greater than 50% were lower than other vaccines but increased in 2022 reaching 16.6% from 13.0%. COVID-19 vaccine had the lowest refusal rates with only 10.6% of physicians reporting rates higher than 50%. For the remaining vaccines, 17.4% of doctors reported refusal rates of 50% or higher (up from 15.8% in 2020).

### Patient education

Patient education and its methods by both cohorts were also explored; results are presented in Table 5. Most physicians communicated recommended vaccines during the visits (68.2% in 2022, up from 65.4%). Other methods such as telephone calls and text messages saw large increases in 2022, 12.8% from 7.0% for the former and 14.6% from 7.4% for the latter. Only 21.8% reported no such communication (down 4 points from 2020). Nearly half of participants across both cohorts reported not using any educational tools when recommending vaccines, with brochures being the most common tool at 34.6% (down from 38.8% in 2020). Posters, videos, websites, and social media had usage rates ranging from one-sixths to one-fifths of surveyed physicians.

**Table 5:** Patient education.

**How do you or your healthcare center communicate the recommended vaccines to the patients?**			**Which educational tools do you use or share with your patient when recommending a vaccine?**		
**Option**	**2020**	**2022**	**Option**	**2020**	**2022**
During visits.	65.4%	68.2%	Brochures	38.8%	34.6%
Through telephone calls.	7.0%	12.8%	Posters	17.6%	17.8%
Through text messages.	7.4%	14.6%	Videos	9.6%	13.2%
Through email.	6.8%	8.0%	Websites	19.6%	19.6%
Through posters and brochures.	25.2%	23.6%	Social Media	15.4%	18.0%
We do not communicate the recommended vaccines.	25.8%	21.8%	I do not use any educational tools.	47.2%	48.2%
**How much time would you spend discussing and recommending vaccines to a HEALTHY YOUNG ADULT who needs them but is NOT WORRIED?**					
**Cohort**	1 to 2 min	3 to 4 min	5 min or more	No time	
**2020**	33.2%	31.8%	18.0%	17.0%	
**2022**	38.2%	27.2%	13.4%	21.2%	
**How much time would you spend discussing and recommending vaccines to a HEALTHY YOUNG ADULT who needs them and is ACTIVELY SEEKING them?**					
**Cohort**	1 to 2 min	3 to 4 min	5 min or more	No time	
**2020**	31.6%	29.4%	21.4%	17.6%	
**2022**	43.6%	24.4%	21.2%	10.8%	
**How much time would you spend discussing and recommending vaccines to a 65-year-old man with CHRONIC CONDITIONS who needs them?**					
**Cohort**	1 to 2 min	3 to 4 min	5 min or more	No time	
**2020**	9.2%	21.4%	57.4%	12.0%	
**2022**	19.4%	30.2%	40.4%	10.0%	

Physicians were also asked about the time they would dedicate to vaccine discussions and recommendations for three hypothetical patients who need vaccines: a healthy young adult who is not worried, a healthy young adult who is actively seeking them, and a 65-year-old man with chronic conditions. For the first patient, 38.2% would dedicate 1–2 min (up from 33.2%) with another 27.2% dedicating 3–4 min (down from 31.8%); however, a fifth would not dedicate any time to such a patient. More physicians would be willing to dedicate time to a patient who is seeking them, with only 10.8% not dedicating any time. Finally, 40.4% would dedicate 5 min or more to the elderly patient (down 17 points from 2020) with another 30.2% dedicating 3–4 min (up from 21.4% in 2020).

## Discussion

### Vaccination attitudes

In this study, the vaccination attitudes of physicians across the UAE were reviewed before and after the introduction of the COVID-19 vaccines. With a total of 1000 responses collected over two iterations from across the UAE, the results show generally an overwhelmingly positive attitude and trust towards vaccines, their efficacy and importance. However, digging deeper, it becomes clear that physicians are facing a number of issues with their patients, specifically with regards to proving, communicating and assuring them of the vaccines’ safety and effectiveness. More than half of the physicians reported a positive change of attitudes during the pandemic and 90% support national campaigns to promote and encourage vaccination. These results echo some of those seen in the literature globally. In an Italian Healthcare Workers (HCWs) study, 88% were found to be favourable to vaccination [21]. Similarly, a French physician study found that 94% of hospital staff were favourable to vaccination [33]as well as a Spanish GPs with highly positive attitudes to elderly vaccination [25].

However, the picture is not complete because in all of these studies, these attitudes were coupled with other contradictory attitudes or practices. Pelullo et al. also found that only 17.7% of the surveyed HCWs always recommended vaccines to their patients [21]). Similarly, another HCW study in 2020 found only 56.8% of staff confident about the effectiveness of vaccines even though 84.7% considered vaccines indispensable tools [18]. In Verger’s study, 40% of the physicians still showed moderate vaccine hesitancy, mainly being worried and unclear about safety; those physicians who showed hesitancy were less likely to recommend vaccines to patients [33]. Even the Spanish GPs had slight scepticism when it comes to safety and efficacy of elderly vaccination schedules [25]. Riccio et al., found an overall inadequate perception regarding the dangerousness and risk of some VPDs with 20% believing that the risks of vaccination outweigh the benefits [26].

In this study, such contradictory attitudes and practices emerged. Vaccine recommendation rates hovered around 60% and reached as low as 30% and 40% for some vaccines. A number of physicians cited either a lack of awareness regarding the recommendations or a lack of responsibility for promoting the various vaccines. Even more, influenza was regarded as a harmless disease by nearly a tenth of the participants. Such a pattern of attitudes and views has become common across the vaccination research literature. In their review, Pavlovic reviewed forty-five studies regarding vaccine effectiveness perceptions by HCPs and found it to be one of the most commonly cited barriers. The majority of vaccine attitudes focused on influenza and found that again, the perceived severity of the illness guided vaccination attitudes and by extension practices [20]. In a Dutch study, 37.6% of General Practitioners listed the severity of a disease as the most important factor for recommending adult vaccines [12]. Similarly, a number of European studies have shown scepticism regarding whether vaccines’ benefits outweigh the risks, with 14.7% of Italian and 29.8% of Slovenian HCPs believing that influenza vaccine’s risks could outweigh its benefits [4], [31].

### Vaccination practices

As for practices, the study highlighted clear gaps in preventative and vaccination practices overall, as well as when it comes to patient education. As would be expected, family medicine doctors showed the highest utilisation of preventative practices explored, yet even they had low rates of vaccination practices with nearly half of them not regularly evaluating the need for adult vaccination, including influenza. In fact, multivariate analysis showed that compared to other specialities, both family medicine and internal medicine did not display significantly higher levels of general immunization status evaluation. Additionally, deficits in other preventative services were clear, from alcohol screening with family physicians to cancer screening overall. No significant differences were seen between the two cohorts, a result which extends to most of the practices’ results. This is the case even when more than half reported having patients with non-COVID-19 VPDs. Additionally, the surveyed physicians did not view adult vaccination as a general physician duty, rather attributing it to family physicians and internists mostly and taking a more supportive role, believing other physicians to do the same. While reporting difficulties evaluating immunisation status, physicians reported using a variety of methods to determine vaccination status and patient education. Other difficulties were also faced when recommending vaccines with the doctors reporting high refusal rates of the various vaccine families. Finally, the lack of time for vaccine education was clear, with the majority dedicating 1–2 min, if any, to healthy young adults for vaccine discussions.

It is important to address the systemic barriers to implementing vaccination practices, whether it be through the adoption of adult vaccination standards (four standards proposed by the National Vaccine Advisory Committee) or the involvement of nurses, medical assistants and other healthcare professionals [9]. Physicians commonly report being stretched for time and having limited resources to be able to effectively counsel patients regarding vaccination and address their concerns [25]. Yarnall et al. showed that to satisfy the preventive recommendations set forth by the United States Preventive Services Task Force, physicians would need 7.4 h per working day [36]. In contrast to this study, where physicians depended mostly on face-to-face discussions, Pizzini et al. found that nearly half of surveyed Italian GPs informed patients about vaccines through visits, followed by posters [22].

In a study of Swiss and French Primary Care Physicians preventive practices, influenza immunisation was the least frequently reported practice at 37% (compared to 99% screening for blood pressure and 95% for smoking) [28]. Moreover and similar to this study, a number of preventive practices, especially related to alcohol use and some cancers are not performed regularly [28]. Similarly, Hungarian physicians also showed the most missed opportunities when it came to influenza immunisation, though preventive services were underutilised across the board [27]. Redondo et al. looked at the practices of Spanish GPs and found that the physicians spend minimal percentages of their consultation time on recommending vaccines, with influenza receiving double the attention usually compared to pneumococcal vaccines. However, they reported greater engagement with vaccination post the COVID-19 pandemic [25].

## Recommendations

The results of this study highlight a number of possible action points. First, while physicians have outright overwhelmingly positive attitudes, deeper digging highlights a level of vaccine hesitancy, whether it be doubts regarding safety, effectiveness, or necessity or a general level of complacency and lack of ownership regarding patients’ vaccination status. Early during the COVID-19 pandemic, it was already recognized that vaccine hesitancy would significantly affect the uptake of the developed vaccines, with the earliest studies showing acceptance rates as low as 30.4%. However, even among those studies, health workers were still considered the most trusted source of guidance regarding COVID-19 vaccines [15]. In order for physicians to be able to communicate vaccines’ safety and effectiveness clearly, they need to be confident about the safety and effectiveness themselves. Multiple studies have shown that clear recommendation and information from an HCP is the most important factor determining the decision to vaccinate [32].

Hence, promoting and cultivating a culture of adult vaccination is essential as patients who remain susceptible to VPDs, especially to paediatrics-dominant illnesses such as measles and rubella, can suffer much more severe outcomes, reaching eight-times what would have been expected in a pre-vaccination era [7]. Yet even at times when physicians may be motivated to promote vaccines, they may be met with severe vaccine hesitancy from the patients. In this study, more than a fifth of participants reported refusal rates of 50% and higher for some vaccines. However, local HCPs had reported receiving little training on how to address vaccine hesitancy among patients and felt unable to engage with vaccine-hesitant patients [6]). As such, further work can also look at the effectiveness of dialogue-based resources in supporting the physicians improve their vaccination practices given their wide-spread usage in other countries [10]; such resources will also need translation into other more commonly used languages locally such as Arabic, Urdu and Hindi.

### Validity

It is important to consider the various limitations of a research. The sampling method adopted might introduce bias in participant selection; however, care was taken to try and minimize this bias by targeting various health authorities, departments, and levels of training. In this study, the attitudes and practices were self-reported and not verified through observation or review. However, the anonymous nature of the questionnaire possibly limits the possibility of social desirability bias among others. Finally, no clear metrics were used to objectively evaluate and quantify vaccine practices; such results can be generated by dedicated studies supported by data from national registries.

## Conclusion

COVID-19 has highlighted the current underutilization of vaccines and the complacent culture that has plagued adult vaccination systems globally, regionally, and even locally. While it is clear that physicians have overly positive attitudes, their practices reflect a more superficial appreciation of vaccines underpinned with scepticism and lack of initiative. It is essential that more physicians adopt a pro-vaccine stance, a feat that can only be done when armed with the proper tools and the right mentality and beliefs.

**Ethics approval and consent to participate**

This study was reviewed and approved by the Research Ethics Committee at the University of Sharjah (Reference Number: REC-20-04-09-01-S). It was conducted in accordance with all relevant guidelines and regulations. Informed consent was obtained from all participants.

**Availability of data and materials**

The datasets used and/or analysed during the current study are available from the corresponding author on reasonable request.

**Funding**

The authors received no financial support for the research, authorship and/or publication of this article.

Uncited references.

.

## CRediT authorship contribution statement

**Hiba J. Barqawi:** Conceptualization, Methodology, Data curation, Writing – review & editing, Supervision, Writing – original draft. **Kamel A. Samara:** Writing – review & editing, Validation, Software, Methodology, Investigation, Formal analysis, Conceptualization, Writing – original draft. **Enad S. Haddad:** Writing – review & editing, Methodology, Data curation. **Layane M. Bakkour:** Writing – review & editing, Methodology, Data curation. **Firas B. Amawi:** Writing – review & editing, Supervision, Conceptualization.

## Declaration of competing interest

The authors declare that they have no known competing financial interests or personal relationships that could have appeared to influence the work reported in this paper.

## Data Availability

Data will be made available on request.
