# Supplementary material for: Attitudes and practices to adult vaccination among physicians before and after COVID-19 pandemic in the United Arab Emirates
Source: Vaccine X. 2024 Feb 1;17:100455. doi: 10.1016/j.jvacx.2024.100455 (PMC10865396; doi:10.1016/j.jvacx.2024.100455)
Supplement: Supplementary data 1 [file mmc1.docx]

# Supplementary Data

The following is the distribution of responses for the 62 physicians who participated in both 2020 and 2022.

Sex

Female 56% (n=35)

Male 44% (n=27)

Citizenship

Emirati 15% (n=9)

Non-arab 16% (n=10)

Other arab 69% (n=43)

Department

Family medicine 6% (n=4)

Internal medicine 26% (n=16)

Others 35% (n=22)

Pediatrics 18% (n=11)

Health Authority

Dha 27% (n=17)

Doh/haad/seha 32% (n=20)

Mohap 39% (n=24)

Level of Training

Consultant 6% (n=4)

General practitioner 11% (n=7)

Intern house officer 15% (n=9)

Resident 50% (n=31)

Senior specialist 6% (n=4)

Specialist 11% (n=7)

Workplace

Government hospital 76% (n=47)

Primary healthcare 6% (n=4)

Private clinic/hospital 16% (n=10)

Number of patients seen in a week

1 to 19 23% (n=14)

20 to 49 40% (n=25)

50 and above 37% (n=23)

Vaccines are safe.

1 2% (n=1)

2 2% (n=1)

3 24% (n=15)

4 73% (n=45)

Vaccinating adults is important.

1 2% (n=1)

2 5% (n=3)

3 16% (n=10)

4 77% (n=48)

The benefit of all recommended vaccines outweigh their harm.

1 3% (n=2)

2 10% (n=6)

3 23% (n=14)

4 65% (n=40)

There is often a lack of time for advising about vaccines.

1 10% (n=6)

2 13% (n=8)

3 39% (n=24)

4 39% (n=24)

Physicians focus more on treatment rather than prevention.

1 6% (n=4)

2 26% (n=16)

3 27% (n=17)

4 40% (n=25)

Patients refuse recommended vaccines when they are healthy.

1 6% (n=4)

2 27% (n=17)

3 44% (n=27)

4 23% (n=14)

Patients refuse vaccines due to safety concerns.

2 21% (n=13)

3 44% (n=27)

4 35% (n=22)

Patients refuse vaccines due to efficacy concerns.

1 10% (n=6)

2 27% (n=17)

3 35% (n=22)

4 27% (n=17)

Patients refuse vaccines due to cost.

1 52% (n=32)

2 15% (n=9)

3 24% (n=15)

4 10% (n=6)

Patients refuse vaccines if they are not covered under insurance.

1 15% (n=9)

2 15% (n=9)

3 39% (n=24)

4 32% (n=20)

Patients refuse vaccines due to religious concerns.

1 37% (n=23)

2 37% (n=23)

3 19% (n=12)

4 6% (n=4)

Patients refuse a vaccine because they think they will not get the disease.

1 10% (n=6)

2 16% (n=10)

3 44% (n=27)

4 31% (n=19)

Adult vaccination rates are low because vaccines are not legally mandatory.

1 11% (n=7)

2 21% (n=13)

3 39% (n=24)

4 29% (n=18)

Adult vaccination rates are low because vaccines are not covered under some insurance plans.

1 13% (n=8)

2 15% (n=9)

3 44% (n=27)

4 29% (n=18)

There should be more national adult vaccination and awareness campaigns.

1 3% (n=2)

2 3% (n=2)

3 26% (n=16)

4 68% (n=42)

How have YOUR attitudes to vaccination changed during the COVID-19 pandemic?

More negative 6% (n=4)

More positive 53% (n=33)

Neutral 40% (n=25)

How have your PATIENTS' attitudes to vaccination changed during the COVID-19 pandemic?

More negative 23% (n=14)

More positive 47% (n=29)

Neutral 31% (n=19)

Have you had ANY patients with a vaccine preventable disease (OTHER THAN COVID-19) in the last 5 years?

I can't recall. 21% (n=13)

No 23% (n=14)

Yes 56% (n=35)

To what extent does the responsibility of determining the patient's general immunization status fall on you?

1.0 11% (n=7)

2.0 18% (n=11)

3.0 48% (n=30)

4.0 23% (n=14)

How difficult is it to evaluate the patient's immunization status?

1.0 11% (n=7)

2.0 44% (n=27)

3.0 34% (n=21)

4.0 11% (n=7)

How often do you screen your patients for vaccine preventable diseases (OTHER THAN COVID-19)?

1.0 24% (n=15)

2.0 34% (n=21)

3.0 24% (n=15)

4.0 18% (n=11)

How often do you think other doctors screen their patients for vaccine preventable diseases (OTHER THAN COVID-19)?

1.0 27% (n=17)

2.0 44% (n=27)

3.0 21% (n=13)

4.0 8% (n=5)

In a month, how many people would REFUSE the influenza vaccine when you recommend it?

0% to 24% 24% (n=15)

25% to 49% 19% (n=12)

50% to 74% 16% (n=10)

75% to 100% 3% (n=2)

Vaccine does not fall under my department/specialty. 37% (n=23)

In a month, how many people would REFUSE a COVID-19 vaccine when you recommend it?

0% to 24% 39% (n=24)

25% to 49% 15% (n=9)

50% to 74% 10% (n=6)

75% to 100% 3% (n=2)

Vaccine does not fall under my department/specialty. 34% (n=21)

In a month, how many people would REFUSE any non-influenza/ non-COVID-19 vaccine when you recommend it?

0% to 24% 27% (n=17)

25% to 49% 19% (n=12)

50% to 74% 13% (n=8)

75% to 100% 3% (n=2)

Vaccination does not fall under my department/specialty. 37% (n=23)

How much time would you spend discussing and recommending vaccines to a HEALTHY YOUNG ADULT who needs them but is NOT WORRIED?

1 to 2 minutes 42% (n=26)

3 to 4 minutes 21% (n=13)

5 minutes or more 11% (n=7)

No time 26% (n=16)

How much time would you spend discussing and recommending vaccines to a HEALTHY YOUNG ADULT who needs them and is ACTIVELY SEEKING them?

1 to 2 minutes 32% (n=20)

3 to 4 minutes 26% (n=16)

5 minutes or more 19% (n=12)

No time 23% (n=14)

How much time would you spend discussing and recommending vaccines to a 65-year old man with CHRONIC CONDITIONS who needs them?

1 to 2 minutes 21% (n=13)

3 to 4 minutes 27% (n=17)

5 minutes or more 32% (n=20)

No time 19% (n=12)

Did you complete this questionnaire previously in 2020?

Yes 100% (n=62)

On a routine patient encounter, which of the following would you do? - Measure patient's blood pressure.

False 26% (n=16)

True 74% (n=46)

On a routine patient encounter, which of the following would you do? - Evaluate patient's smoking status.

False 50% (n=31)

True 50% (n=31)

On a routine patient encounter, which of the following would you do? - Measure patient's weight.

False 42% (n=26)

True 58% (n=36)

On a routine patient encounter, which of the following would you do? - Evaluate patient's influenza immunization status.

False 74% (n=46)

True 26% (n=16)

On a routine patient encounter, which of the following would you do? - Evaluate patient's general immunization status.

False 53% (n=33)

True 47% (n=29)

On a routine patient encounter, which of the following would you do? - Evaluate if patient was screened for colorectal cancer, when relevant.

False 77% (n=48)

True 23% (n=14)

On a routine patient encounter, which of the following would you do? - Evaluate if male patient was screened for prostate cancer, when relevant.

False 81% (n=50)

True 19% (n=12)

On a routine patient encounter, which of the following would you do? - Evaluate if female patient was screened for breast cancer, when relevant.

False 77% (n=48)

True 23% (n=14)

On a routine patient encounter, which of the following would you do? - Evaluate if female patient was screened for cervical cancer, when relevant.

False 79% (n=49)

True 21% (n=13)

On a routine patient encounter, which of the following would you do? - Evaluate patient's alcohol intake.

False 68% (n=42)

True 32% (n=20)

On a routine patient encounter, which of the following would you do? - Evaluate patient's lipid profile.

False 65% (n=40)

True 35% (n=22)

Who should be responsible for determining the patient's immunization status? - Internal Medicine doctors

False 60% (n=37)

True 40% (n=25)

Who should be responsible for determining the patient's immunization status? - Family Medicine doctors

False 8% (n=5)

True 92% (n=57)

Who should be responsible for determining the patient's immunization status? - Emergency doctors

False 77% (n=48)

True 23% (n=14)

Who should be responsible for determining the patient's immunization status? - Obstetricians and Gynecologists

False 84% (n=52)

True 16% (n=10)

Who should be responsible for determining the patient's immunization status? - Nurses

False 73% (n=45)

True 27% (n=17)

Who should be responsible for determining the patient's immunization status? - Pharmacists

False 94% (n=58)

True 6% (n=4)

Which of the following do you use to evaluate the patient's general immunization status? - I verbally ask the patient.

False 32% (n=20)

True 68% (n=42)

Which of the following do you use to evaluate the patient's general immunization status? - Other medical staff ask the patient.

False 84% (n=52)

True 16% (n=10)

Which of the following do you use to evaluate the patient's general immunization status? - I check the patient's file.

False 37% (n=23)

True 63% (n=39)

Which of the following do you use to evaluate the patient's general immunization status? - I check the patients immunization records.

False 56% (n=35)

True 44% (n=27)

Which of the following do you use to evaluate the patient's general immunization status? - I do not determine the immunization status of my patients.

False 90% (n=56)

True 10% (n=6)

How do you or your healthcare center communicate the recommended vaccines to your patients? - During visits.

False 40% (n=25)

True 60% (n=37)

How do you or your healthcare center communicate the recommended vaccines to your patients? - Through telephone calls.

False 89% (n=55)

True 11% (n=7)

How do you or your healthcare center communicate the recommended vaccines to your patients? - Through text messages.

False 92% (n=57)

True 8% (n=5)

How do you or your healthcare center communicate the recommended vaccines to your patients? - Through email.

False 94% (n=58)

True 6% (n=4)

How do you or your healthcare center communicate the recommended vaccines to your patients? - Through posters and brochures.

False 74% (n=46)

True 26% (n=16)

How do you or your healthcare center communicate the recommended vaccines to your patients? - We do not communicate the recommended vaccines.

False 82% (n=51)

True 18% (n=11)

Which educational tools do you use or share with your patient when recommending a vaccine? - Brochures

False 56% (n=35)

True 44% (n=27)

Which educational tools do you use or share with your patient when recommending a vaccine? - Posters

False 76% (n=47)

True 24% (n=15)

Which educational tools do you use or share with your patient when recommending a vaccine? - Videos

False 81% (n=50)

True 19% (n=12)

Which educational tools do you use or share with your patient when recommending a vaccine? - Websites

False 79% (n=49)

True 21% (n=13)

Which educational tools do you use or share with your patient when recommending a vaccine? - Social Media

False 90% (n=56)

True 10% (n=6)

Which educational tools do you use or share with your patient when recommending a vaccine? - I don't use any educational tools.

False 65% (n=40)

True 35% (n=22)
